# Epidemic versus endemic West Nile virus dead bird surveillance in California: Changes in sensitivity and focus

**DOI:** 10.1371/journal.pone.0284039

**Published:** 2023-04-06

**Authors:** Leslie Foss, Tina Feiszli, Vicki L. Kramer, William K. Reisen, Kerry Padgett

**Affiliations:** 1 Vector-Borne Disease Section, California Department of Public Health, Richmond, California, United States of America; 2 Vector-Borne Disease Section, California Department of Public Health, Sacramento, California, United States of America; 3 Department of Pathology, Microbiology and Immunology, School of Veterinary Medicine, University of California, Davis, CA, United States of America; University of California Los Angeles, UNITED STATES

## Abstract

Since 2003, the California West Nile virus (WNV) dead bird surveillance program (DBSP) has monitored publicly reported dead birds for WNV surveillance and response. In the current paper, we compared DBSP data from early epidemic years (2004–2006) with recent endemic years (2018–2020), with a focus on specimen collection criteria, county report incidence, bird species selection, WNV prevalence in dead birds, and utility of the DBSP as an early environmental indicator of WNV. Although fewer agencies collected dead birds in recent years, most vector control agencies with consistent WNV activity continued to use dead birds as a surveillance tool, with streamlined operations enhancing efficiency. The number of dead bird reports was approximately ten times greater during 2004–2006 compared to 2018–2020, with reports from the Central Valley and portions of Southern California decreasing substantially in recent years; reports from the San Francisco Bay Area decreased less dramatically. Seven of ten counties with high numbers of dead bird reports were also high human WNV case burden areas. Dead corvid, sparrow, and quail reports decreased the most compared to other bird species reports. West Nile virus positive dead birds were the most frequent first indicators of WNV activity by county in 2004–2006, followed by positive mosquitoes; in contrast, during 2018–2020 mosquitoes were the most frequent first indicators followed by dead birds, and initial environmental WNV detections occurred later in the season during 2018–2020. Evidence for WNV impacts on avian populations and susceptibility are discussed. Although patterns of dead bird reports and WNV prevalence in tested dead birds have changed, dead birds have endured as a useful element within our multi-faceted WNV surveillance program.

## Introduction

West Nile virus (WNV) is a mosquito-borne flavivirus which is maintained in a transmission cycle between ornithophilic mosquitoes and birds. Spillover to humans and other mammals frequently occurs and WNV disease is the most common cause of mosquito-borne disease in the United States [1]. In 1999, when the virus was first detected in New York City (NYC), USA, dead and dying American Crows *(Corvus brachyrhynchos)* were the first indication of this emerging pathogen [2]. Clusters of dead crow reports and numbers of WNV-positive dead crows became effective early warning indicators of WNV activity in specific geographic areas of NYC before corresponding human cases were reported [2, 3]. Many North American birds succumbed to WNV as it spread westward and southward via mosquitoes and seasonal bird migration and dispersal. Consequently, many U.S. and Canadian states and provinces established passive surveillance programs that included dead bird reporting and testing, and these data continued to precede human cases as the virus spread [4–6]. Currently, the reporting and testing of dead birds to monitor WNV activity has declined in most of North America, with public health officials citing reasons such as resource limitations, lower utility, and reduced public participation [7]. In 2016, only 15 of 48 states employed WNV dead bird testing to inform mosquito control efforts to reduce WNV transmission risk [7].

Public health priorities are continually shifting and as a result, the California DBSP has been modified over the years. Previous research focused on summarizing WNV prevalence in dead birds and other statistics since the program’s inception [8, 9]. However, we observed marked changes in program operations and the numbers of dead birds reported and tested when comparing the first few years of the program to the most recent years. We summarized these differences by comparing the early epidemic years (2004–2006) to the most recent endemic years (2018–2020) in the DBSP to characterize changes in: 1) program operations, 2) reports by county and species, 3) WNV prevalence in dead birds, and 4) first environmental detection of WNV. Interim years (2007–2017) were not included (except for Breeding Bird Survey Data), as we were specifically interested in the long-term impact of the virus on dead bird surveillance and bird populations. The overall purpose of our study was to summarize those differences, and then use the findings to prove or disprove two hypotheses: 1. Dead bird reports have decreased in later years, due in part to decreases in bird populations, and 2. Despite the length of time that WNV has been circulating in California, some bird species remain susceptible to dying from the virus. These hypotheses emerged from the most prominent questions surrounding dead bird surveillance program trends.

### Dead bird surveillance program history

West Nile virus was added to California’s Arbovirus Surveillance Program in 2000 [10–12] in anticipation of the arrival of WNV from the East. This comprehensive program is a collaboration among the California Department of Public Health (CDPH), the University of California, Davis Arbovirus Research and Training (DART) laboratory [formerly the Center for Vector-borne Diseases, CVEC], and local vector control agencies (hereafter VCAs) and public health agencies throughout California.

In 2003, WNV was first detected in Southern California in *Culex tarsalis* mosquitoes, followed by dead birds, sentinel chickens, and three human cases [11]. By the end of 2004, WNV had been detected in dead birds from all 58 California counties [13], and they were the only indicator of WNV activity in 23 counties.

In the early years (~2004–2006), a few VCAs (usually supported by UC Davis) trapped wild rural and peridomestic birds for sera collection and antibody testing to understand WNV epidemiology and perhaps enhance virus detection [11, 14–17]. Although useful for identifying avian hosts and showing trends in virus amplification and avian flock immunity, these data were confounded by the inability to detect date and place of infection and therefore were less useful for tracking human risk [18]. In contrast, the DBSP provides passive surveillance of WNV transmission activity by detecting birds that recently succumbed from infection. However, this program relies upon public participation to locate and report avian carcasses and therefore is biased by variation in human population density, avian species abundance, distribution and susceptibility to infection, virus evolution and transmission dynamics, and intervention. Because of their large size, urban distribution, and high susceptibility to lineage I WNV infection, surveillance has tended to focus on American crows, especially in the Los Angeles Basin and Sacramento areas where this species is abundant [19].

### Dead bird reports

To monitor avian mortality due to WNV, CDPH maintains a WNV and Dead Bird Call Center (formerly “hotline”) (1-877-WNV-BIRD) and website [20], launched in 2002 in anticipation of the arrival of WNV in California [21]. Through a variety of outreach strategies, the public is encouraged to report dead birds to the DBSP. Initially all reports were made by phone to the call center, but in 2006 an online reporting option was added. Call center staff review reports to determine the likely species and condition of the bird. Reports are entered into a SQL Bird Information Reporting Database (B.I.R.D.) which integrates with a centralized database for all environmental data [22] and geocodes each report. In addition, since 2013, smart AI algorithms have triaged incoming online reports based on specific parameters in the B.I.R.D. database such as the presence of insects and age of the carcass. Individuals who report dead birds online receive immediate email replies with recommendations for their dead bird (e.g., the bird is suitable for testing and staff will arrange pickup). If a dead bird is deemed suitable for testing, call center staff call and ask the reporting individual to safely secure it, and the report is submitted to the appropriate VCA for collection and WNV testing. The types of bird species a VCA collects may change mid-season or from one year to the next depending on local resources and the likelihood of detecting WNV with a limited versus broader list of accepted species [23, 24]. Outreach campaigns by VCAs and CDPH provide mosquito bite and control messages to the public, with some emphasizing dead bird reporting. Popular social media platforms have made it easier to broadcast messages through engaging videos and graphics [25].

Since the DBSP relies on the public to report dead birds, the number of reports is often a gauge of both WNV activity and public awareness and participation. Initially, the high numbers of dead bird reports were useful to identify hotspots of WNV activity [2, 26, 27]. However, in more recent years, WNV test results from dead birds are used due to fewer reports and frequent non-WNV mortality in birds [28, 29]. Statewide and regionally, dead bird reports have fluctuated over the years [7], but rural areas consistently have contributed fewer dead bird reports than urban areas, partly due to lower density of people to find carcasses and in some regions, due to fewer corvid species and lesser abundance [30, 31].

### West Nile virus testing

Testing protocols also have been modified over the years. Beginning in 2003, dead birds were shipped on ice packs to the California Animal Health and Food Safety (CAHFS) laboratory for final species identification and tissue collection. Dead birds were identified according to the American Ornithologists’ Union [32]. Kidney tissue or oral swab samples were sent to DART for RT-qPCR or Vero cell culture testing [11, 33]. Later, rapid antigen tests of avian oral swabs were evaluated and added as a testing option not requiring necropsy: VecTest (Medical Analysis Systems Camarillo, CA, USA) [34, 35] and Rapid Analyte Measurement Platform (RAMP, Response Biomedical Corp, Burnaby, British Columbia, Canada) [35]. Results from these tests were evaluated in parallel with RT-qPCR results [13], and later proficiency panels were developed and issued annually by DART for standardization (2006–2018) [34, 36]. Beginning in 2013, protocols shifted to oral swabs only (collected by VCAs) pressed onto nucleic acid preservation cards and mailed to DART for RT-qPCR testing [37]. Since carcasses were no longer being shipped to CAHFS, VCAs were primarily responsible for identifying dead birds to species after triage by CDPH. Over the years, some VCAs developed internal laboratory capacity to test dead birds (RT-qPCR of various tissues or oral swabs) and completed annual proficiency panels issued by DART to ensure testing standardization [12].

### Important indicator species

American Crows have been the most frequent species reported to the DBSP, and along with other corvid species such as Common Ravens *(Corvus corax)*, California Scrub-jays *(Aphelocoma californica)* and Yellow-billed Magpies *(Pica nuttalli)*, are good indicators of WNV activity because they develop high WNV viremias and succumb soon after infection [38–40]. Other species such as House Finches *(Haemorhous mexicanus*), House Sparrows *(Passer domesticus)*, and American Robins *(Turdus migratorius)* may develop high viremias but suffer low to moderate mortality [41, 42]. These species (as well as American Crows) exploit urban and suburban landscapes for food, nesting, and predator avoidance, resulting in low avian diversity consisting of many competent avian hosts. Such conditions are suitable for WNV amplification and human infection from *Culex spp*. mosquitoes [43]. Raptors such as Cooper’s Hawks *(Accipiter cooperii)* and Red-tailed Hawks *(Buteo jamaicensis)* are also susceptible to WNV and frequently test positive in the DBSP [8, 44]. Mourning Doves *(Zenaida macroura)*, as well as other doves, quail, and pigeons, are commonly reported in the DBSP but are not usually tested, because they survive infection and therefore are not ideal indicators of WNV activity [45, 46]. Nonetheless some VCAs test Mourning Doves and occasionally detect WNV [9]. Although reports from all bird taxa are collected, certain species are the focus of this study for their susceptibility to infection and value as indicators of WNV activity [38].

### WNV prevalence in dead birds

The prevalence of WNV in dead birds has fluctuated statewide and by county over the years, from a high of 59.4% in 2014 [9] to a low of 12% in 2019 [47]. West Nile virus prevalence in a particular bird species is a function of the local avifauna composition and the number of birds reported by the public, picked up by VCAs and testing positive. The strain of WNV circulating has changed over time and has influenced mortality in birds: the WNV NY99 strain (introduced to the U.S. in 1999) caused elevated viremia and mortality in American Crows [48] as well as House Sparrows [49], but this strain was eventually displaced with strains WN02 and SW03 which have higher replicative fitness in House Finches and *Culex tarsalis* mosquitoes [50], but have retained the important T249P amino acid substitution responsible for high morality in corvids [51]. Strains WN02 and SW03 remain the dominant strains of WNV in California today [52]. The prevalence of WNV in the most frequently tested species was described previously [8, 9]. This species profile appears unchanged from early to late years. A closer look at WNV prevalence in those species over time, however, may provide clues about evolving avian susceptibility.

### First environmental indicator of WNV in county

As WNV surveillance programs were established across the U.S., surveillance elements, including dead birds, mosquitoes, and sentinel chickens, were evaluated as early indicators of viral activity over the course of the WNV transmission season, typically late spring through early fall. WNV-positive dead birds preceded human cases not only in afore-mentioned NYC [2] but also in Colorado, where they were the most accurate surveillance element at predicting human cases [53]. In California, positive dead birds were the first indicators of WNV activity in 90% of counties in 2004 [54]. American Crows were found to amplify WNV in localized areas of Southern California where human cases later emerged [55]. A study of California’s program during 2004–2012 found dead bird reports detected WNV activity ~1–2 weeks prior to a sentinel chicken seroconversions and concordant with early WNV-positive mosquitoes [24]. In the early years, dead birds were tested year-round by most districts before WNV activity patterns in California were determined. In recent years, only a few districts continued to test dead birds throughout the winter months, as WNV activity typically ends by November and does not pick up again until spring (CDPH unpublished data). West Nile virus positive dead birds that were detected during the winter months often had low-level infections [56] likely not related to recent transmissions or human disease risk [9]. These winter detections were thought to be persistent chronic infections [56] and are therefore not included in our current study. Whether or not birds remain effective as early indicators of the resumption of WNV activity is one aim of the current study.

## Methods

### Ethics statement

Enzootic surveillance data are owned by the agencies that generated them; permission to use the data was obtained through CalSurv data request #000062 submitted to the California Vector-borne Disease Surveillance System. Permission to salvage dead birds for WNV testing was granted by the California Department of Fish and Wildlife through a memorandum of understanding which is renewed every five years [12].

### Data sources

Data were gathered from several sources for summarization, analysis, and evaluation against the hypotheses. Dead bird data was obtained from Microsoft Access databases (2004–2005) and the B.I.R.D. database (2006–2020) which are managed by CDPH and the CalSurv system at UC Davis [12]. Bird abundance data from 2004 to 2019 were obtained from Breeding Bird Survey (BBS), a longstanding, robust citizen science dataset available online [57] (data for 2020 were not available). The BBS designates various Bird Conservation Regions and provides data based on those regions. California overlaps five Bird Conservation Regions (BCR), but the majority of WNV activity occurs in three BCRs: the Coastal region (which includes the Coast from the Bay Area south, the inland Central Valley, and Southern California), and to a lesser extent, the Sierra Nevada Mountain region in the east and the Sonoran and Mojave Deserts region in the southeast. To better understand population trends in various parts of California, we not only gathered relative abundance trends in key species for WNV ecology overall in California, but also within these three BCRs from 2004 to 2019.

### Comparisons

Comparisons of the following data were made between early (2004–2006) and recent (2018–2020) periods for:

- **Collection criteria:** Criteria for dead bird collection and testing.
- **County report incidence:** Report incidence for each time period was calculated by multiplying the number of reports from each county by 100,000 and dividing by each county’s mean human population in either the early time period or the later time period. County population estimates were obtained from the Department of Finance website [58].
- **County human WNV incidence:** Number of WNV cases in each county, multiplied by 100,000 and divided by the mean county population in either the early period or the late period.
- **Species reported**: Numbers of each taxa (some by categories such as raptors) were totaled. Selected species such as corvids and house finches were totaled separately because these taxa play a key role in WNV ecology.
- **West Nile virus infection prevalence:** Determined for key avian species as number positive for WNV antigen or RNA divided by the number tested.
- **First environmental indicator of WNV in a county:** First detection of WNV in each county by dead birds, mosquitoes, or sentinel chickens was listed for 2005 and 2020, years representative of early epidemic and recent enzootic periods. The year 2005 was chosen for the early period because the dead bird program was solidly in place by that time. First positive detections prior to disease weeks 14 (mid-April) were considered winter (or pre-WNV season) detections and were excluded.
- **Species abundance:** Breeding Bird Survey (BBS) data from 2004 to 2019 for important species were evaluated for changes in population abundance (data for 2020 were not yet available). We used annual abundance indices to help eliminate observer bias [59].

### Statistics

Chi-square tests were conducted using Microsoft^©^ Excel on differences in species composition within reports and differences in WNV prevalence in 2004–2006 compared to 2018–2020.

## Results

### Dead bird surveillance program operations

#### Collection criteria

Parameters (e.g., species, condition) for carcass submission were compared for 2005 and 2020 [S1 Table]. In 2005, 82 (92%) of 92 VCAs collected all bird species except doves, quail, and pigeons for WNV testing. Birds must have been dead for less than 24h with no evidence of trauma or decay (maggots/sunken eyes). In contrast, by 2020, only 53 (60%) of 88 VCAs (four were absorbed into other VCAs) collected birds for WNV testing and many limited the variety of species selected. In 2020, 30 VCAs still collected most bird species for testing, whereas 9 VCAs collected only corvids, raptors, and songbirds (defined in the program as other perching birds besides corvids), 9 VCAs collected corvids only, 4 VCAs collected only corvids and raptors, and 1 VCA collected crows and ravens only. Shorebirds, waterbirds, turkeys, peafowl, and vultures were commonly excluded because they had rarely tested WNV positive in previous years. Increasingly, some VCAs began collecting birds that had been dead up to 48h instead of 24h as it was determined WNV could still be detected in older carcasses (CDPH unpublished data). By 2020, 15 VCAs had adopted the 48h criterion, increasing the number of suitable birds for testing [S1 Table]. Among those 15 VCAs, 3 collected carcasses of any age and condition because methods had been developed to test samples from old carcasses [60, 61].

#### Dead bird reporting and county incidence

In 2020, approximately half of the dead bird reports were received online, which reduced the number of staff needed to process reports. Recent dead bird report totals were much lower compared to early years; in 2005, more than 100,000 dead bird reports were received [62], while in 2020 under 6,000 were received [47].

A total of 223,855 reports were received in 2004–2006 compared to 19,859 received in 2018–2020, a 91% decrease. Of the top ten counties with the most dead bird reports in 2004–2006, five remained among the top ten counties in 2018–2020 (Los Angeles, Sacramento, Fresno, Contra Costa, and Santa Clara) (Table 1). Four counties with high numbers of dead bird reports in the early years (San Bernardino, Riverside, Stanislaus, and San Joaquin) were replaced with two Bay Area counties (Alameda and San Mateo), one southern California county (Orange), and one northern Central Valley county (Yolo) in recent years (Table 1). Human WNV incidence also decreased overall from the early to the later time period.

**Table 1:** Top ten California counties in early and recent time periods: Total number of reports, report incidence, and corresponding WNV human incidence for each three-year period.

	Years 2004–2006				Years 2018–2020			
	County	# Reports	Report incidence*	WNV Human incidence*	County	# Reports	Report incidence*	WNV Human incidence*
1	Los Angeles	33,599	343	4.02	Sacramento	2,989	192	1.67
2	Sacramento	25,121	1,850	14.44	Los Angeles	2,395	24	1.61
3	Fresno	12,911	1,479	10.89	Orange	1,673	52	0.97
4	San Bernardino	12,531	646	12.11	Santa Clara	1,628	84	0.1
5	Contra Costa	11,528	1,148	1.89	Contra Costa	1,533	133	0.78
6	Riverside	10,397	537	11.57	Alameda	936	56	0.06
7	Stanislaus	9,421	1,894	20.91	San Mateo	886	115	0
8	San Joaquin	8,293	1,275	7.23	Placer	787	198	3.02
9	Santa Clara	7,925	465	0.65	Fresno	700	69	7.42
10	Placer	7,212	2,307	14.08	Yolo	645	293	7.27
Totals		138,938				14,172		

*Per 100,000 persons/3 year mean

#### Report incidence

Counties with large human and American Crow populations tended to generate the most reports, but this was not true for every county in the top ten list (Table 1). (For American Crow range in California, see [19]). The most reports came from Los Angeles County in 2004–2006, the most populated county in California in 2006 with approximately 10 million people. In 2018–2020 the most reports came from Sacramento County, which was the 8th most populated county in California in 2020 with 1.6 million people. Eight counties from the top ten list in either early or recent years had population estimates of about one to three million (Alameda, Contra Costa, Fresno, Orange, Riverside, San Bernardino, Sacramento, and Santa Clara); four counties had a population of 500,000–950,000 (San Mateo, Stanislaus, and San Joaquin); and two had a population of 200,000–400,000 (Placer and Yolo). Although San Diego County has a population of about 3.1 million, it was not one of the top ten counties for either period. Report incidence in the early period was highest in Placer County (2,307 reports per 100,000 persons), followed by Stanislaus (1,894) and Sacramento (1,850) counties (Table 1). In the later period, report incidence was lower overall, with the highest report incidence in Yolo County (293), followed by Placer (198) and Sacramento (192) counties (Table 1).

The number of dead bird reports decreased in every county from the early to recent periods [S2 Table]. In 14 counties the report total decreases were less than the average decrease (93%; overall decrease: 91%), especially for San Mateo (68% decrease), Orange (74%), and Santa Clara (79%) counties.

#### Species reported

The number of dead corvids reported (with percentage of all reports in parentheses) decreased from 112,143 (50.23%) to 6,883 (34.66%) (Table 2). Dead sparrow reports also decreased from 24,731 (11.05% of all reports) to 962 (4.84%). Percentages also decreased in three other categories (psittacine, other exotic spp., and other native spp.), but only slightly, and these report number differences were not significant (Table 2). However, quail percentages decreased from the early to the later period, and the numerical decrease was significant (p = 0.03). For all other types of birds, their percentages increased in the later period, as might be expected due to the overall decrease in reports.

**Table 2:** Dead bird report totals by category and percentages of total reported birds, early versus later years. Unshaded categories increased in proportion while shaded species decreased.

Category	# Dead bird reports (2004–2006)	# Dead bird reports (2018–2020)	Percent of total (2004–2006)	Percent of total (2018–2020)	P*
Passerine (perching birds)					
Corvid	112,443	6,883	50.23	34.66	<0.0001
Finch	8,813	921	3.94	4.64	0.00012
Sparrow	24,731	962	11.05	4.84	<0.0001
Thrush	3,898	540	1.74	2.72	<0.0001
Other songbird	11,759	2,703	5.25	13.61	<0.0001
Raptor—hawk, falcon, vulture	2,863	993	1.28	5.00	<0.0001
Raptor—owl	1,652	433	0.74	2.18	<0.0001
Hummingbird	1,249	225	0.56	1.13	<0.0001
Woodpecker	1,134	195	0.51	0.98	<0.0001
Duck or goose	1,170	160	0.52	0.81	<0.0001
Waterbird	151	75	0.07	0.38	<0.0001
Seabird	434	135	0.19	0.68	<0.0001
Shore/wading bird	487	169	0.22	0.85	<0.0001
Pigeon or dove	19,024	2,156	8.50	10.86	<0.0001
Quail	811	53	0.36	0.27	0.0302
Chicken or fowl	1,063	172	0.47	0.87	<0.0001
Psittacine (parrot family)	794	66	0.35	0.33	0.611
Other spp. (exotic)	269	15	0.12	0.08	0.077
Other spp. (native)	144	7	0.06	0.04	0.115
Unknown spp.	30,966	2,996	13.83	15.09	<0.0001
Totals	223,855	19,859	100.00	100.00	

*Chi-square: report number difference between periods, compared to the overall report difference.

#### Corvid reports

Dead corvid reports were further divided by species (Table 3). Reports decreased notably for all species, but least for Common Ravens. Chi-square tests comparing report numbers between time periods were significant (all p<0.0001). The percentages of American Crow and Common Raven reports among all corvid reports increased (by 13% and 3%, respectively), whereas percentages of all other corvid reports decreased (<1%-6%) from the early to the recent time periods. The category “other corvids” included Black-billed Magpie *(Pica hudsonia)*, Clark’s Nutcracker *(Nucifraga columbiana)*, Pinyon Jay *(Gymnorhinus cyanocephalus)*, Island Scrub-Jay *(Aphelocoma insularis)*, Woodhouse’s Scrub-Jay *(Aphelocoma woodhouseii)*, and unspecified “jay” in 2004–2006 data.

**Table 3:** Number of dead corvid reports and percent of total corvid reports, early versus later years. The last column is the overall percent decrease in reports; shaded species decreased proportionally while unshaded species increased proportionally.

Species	# Reports 2004–2006	Percent of total (2004–2006)	# Reports 2018–2020	Percent of total (2018–2020)	% Decrease
American Crow	67,127	59.70	4,984	72.41	93
Common Raven	2,926	2.60	415	6.03	86
Yellow-billed Magpie	10,262	9.13	194	2.82	98
California Scrub-jay	27,812	24.73	1,227	17.83	96
Steller’s Jay	3,623	3.22	54	0.78	99
Other corvids	693	0.61	9	0.13	99
Totals	112,443		6,883		94

### West Nile virus prevalence

#### Infection prevalence in corvids

West Nile virus infection prevalence in dead corvids decreased from early to later years by 21–33% (all p<0.01 except Common Ravens which decreased by 3%; p = 0.306) (Table 4). West Nile virus prevalence most notably decreased in dead Yellow-billed Magpies (Table 4). These data reflected the decrease in enzootic transmission between sampling periods.

**Table 4:** West Nile virus infection prevalence in tested dead corvids, early versus recent years.

	2004–2006				2018–2020		
Species*	# Positive	# Tested	Prevalence (%)	# Positive	# Tested	Prevalence (%)	p
Yellow-billed Magpie	718	934	77	51	115	44	<0.0001
California Scrub-jay	1,543	2,470	62	204	562	36	<0.0001
American Crow	2,829	5,708	50	575	1,970	29	<0.0001
Steller’s Jay	99	227	44	2	18	11	0.007
Common Raven	49	431	11	12	144	8	0.306
Totals	5,238	9,770		844	2,809		

*Other corvid species tested in 2004–2006 included 9/14 positives, and none in 2018–2020 as those species are rarely reported and tested.

#### Infection prevalence in other important species

West Nile virus prevalence in dead finches, sparrows, thrushes (including American Robins), and other songbirds (excluding corvids) decreased by either 3% or 7% from the early to recent periods (Table 5) (all p<0.05). In raptors, the decrease was much greater (e.g., from 19% to 4% in hawks, falcons, and vultures; 19% to 2% in owls) (both p < .0001) (Table 5).

**Table 5:** West Nile virus prevalence in select tested dead birds, 2004–2006 compared to 2018–2020.

	2004–2006			2018–2020			
Species	# Positive	# Tested	Prevalence (%)	# Positive	# Tested	Prevalence (%)	p
Finches	276	1,534	18	47	413	11	0.001
Sparrows	164	1,605	10	24	357	7	0.042
Thrushes	123	705	17	22	219	10	0.008
Other songbirds	238	2,210	11	61	815	7	0.007
Hawks, falcons, vultures etc.	215	809	27	19	515	4	<0.0001
Owls	83	432	19	5	227	2	<0.0001
Totals	1,099	7,295		178	2,546		

#### First seasonal environmental indicator of WNV in county

In 2005, WNV was detected in 54 California counties [62]. In 34 of those counties, WNV was detected by at least two of the three main environmental surveillance elements (dead birds, mosquitoes, and sentinel chickens). Among those 34 counties, dead birds were the first indicator in 27 (79%), mosquitoes were the first indicator in 4 (12%) counties, and sentinel chickens were the first indicator in no counties (Table 6). In three counties (Lake, Riverside, and Solano), a positive dead bird and a positive mosquito sample were found during the same disease week. The average disease week of first WNV detection in dead birds was week 24 (n = 33), in mosquitoes, week 27 (n = 29), and in sentinel chickens, week 30 (n = 31). In nineteen counties, a positive dead bird provided the only WNV detection in 2005 (Table 6), therefore these counties were excluded from this analysis.

**Table 6:** Frequency of first detections of West Nile virus (from disease weeks 14–52) in counties with a detection in at least two of three environmental surveillance elements, and average disease week of first detections. (Due to rounding, total percentages are not 100%).

# Counties with first indicators of WNV				
Year	Birds	Mosquitoes	Sentinel Chickens	Ties
**2005**	27/34 (79%)	4/34 (12%)	0/34 (0%)	3/34 (9%)
**Ave week of first detection**	24	27	30	
**2020**	7/22 (32%)	12/22 (55%)	1/22 (5%)	2/22 (9%)
**Ave week of first detection**	28	27	34	

In 2020, WNV was detected in 40 counties. In 22 of those counties, WNV was detected in at least two of the three main environmental surveillance elements. Among those 22 counties, dead birds were the first indicators in 7 (32%) counties, mosquitoes in 12 (55%) counties, and sentinel chickens in 1 (5%) county (Butte) (Table 6). In Contra Costa County, a positive dead bird and a positive mosquito sample were found in the same disease week, and in Glenn County, a positive mosquito sample and a positive sentinel chicken were found in the same disease week. The average disease week of first WNV detection in dead birds was 28 (n = 17), in mosquitoes it was 27 (n = 22), and in sentinel chickens it was 34 (n = 13). In four counties not included in the analysis, a positive dead bird provided the only WNV detection in 2020 (Alameda, Alpine, Marin, and San Mateo) (Table 6).

#### Breeding Bird Survey statewide trends

Relative abundance of key species for WNV ecology in California 2004–2019 was gathered from BBS data. Selected species showed decreases in population abundance from 2004, the first year of our study, to 2019 (last available year of BBS data) (Table 7). Most corvid species decreased except Common Ravens which increased. Yellow-billed Magpies showed the greatest decrease. Declines were found in the three other perching birds of our focus (American Robins, House Sparrows, and House Finches). Among raptors selected, two hawk species did not appear to change in abundance while Great Horned Owls increased (Table 7).

**Table 7:** Breeding Bird Survey adjusted count trends: Relative abundance change (%) per year in California, 2004–2019. Unshaded species displayed an increase or no change; shaded species a decrease.

Species	Trend Estimate	2.5% CI	97.5% CI
American Crow	-4.94	-6.21	-3.7
Common Raven	3.62	2.28	5.05
Yellow-billed Magpie	-8.33	-10.62	-6.19
California Scrub-jay	-2.04	-2.92	-1.24
Steller’s Jay	-0.97	-1.85	-0.1
American Robin	-0.91	-1.48	-0.32
House Sparrow	-3.05	-4.31	-1.82
House Finch	-1.31	-2.47	-0.15
Red-tailed Hawk	0.37	-0.74	1.43
Cooper’s Hawk	2.12	-0.82	5.12
Great-horned Owl	3.06	0.48	5.79

#### Breeding Bird Survey bird conservation region trends

**Fig 1:**
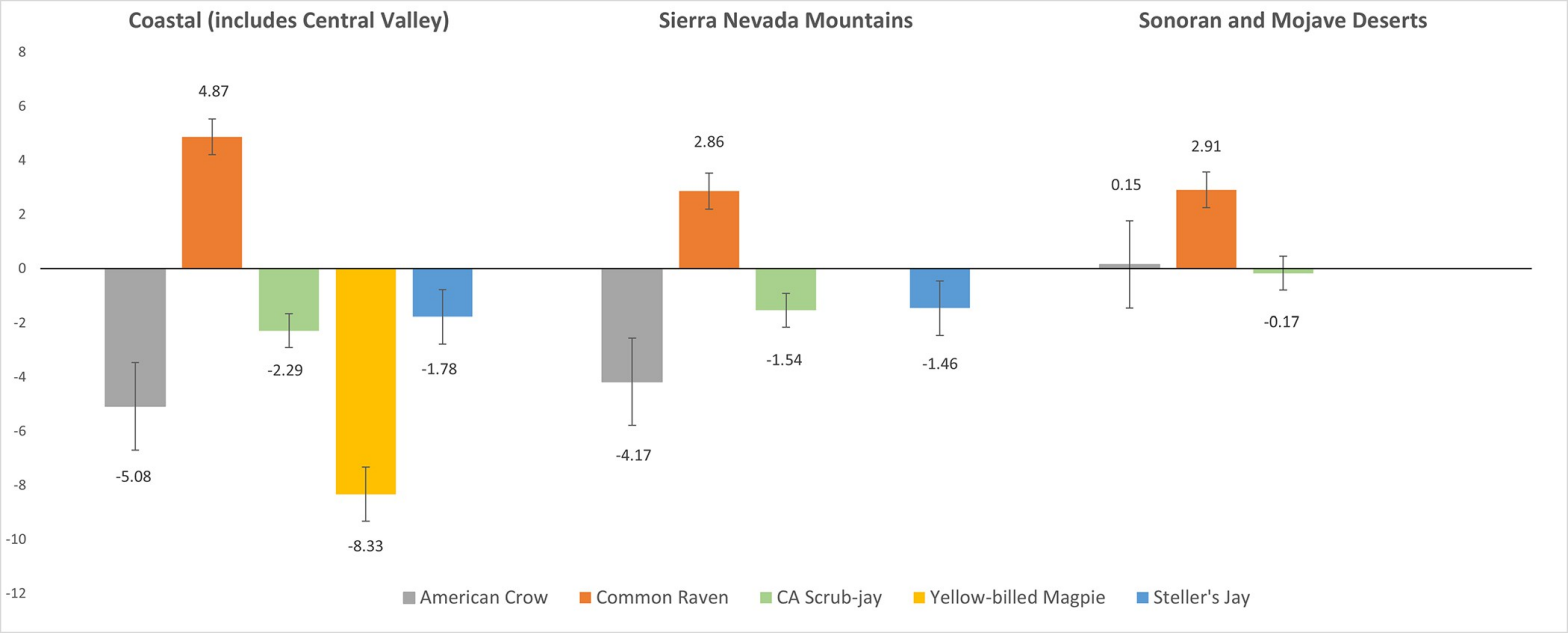
Select corvid species population trends in three Bird Conservation Regions (2004–2019): Relative abundance change (%) per year and standard errors.

**Fig 2:**
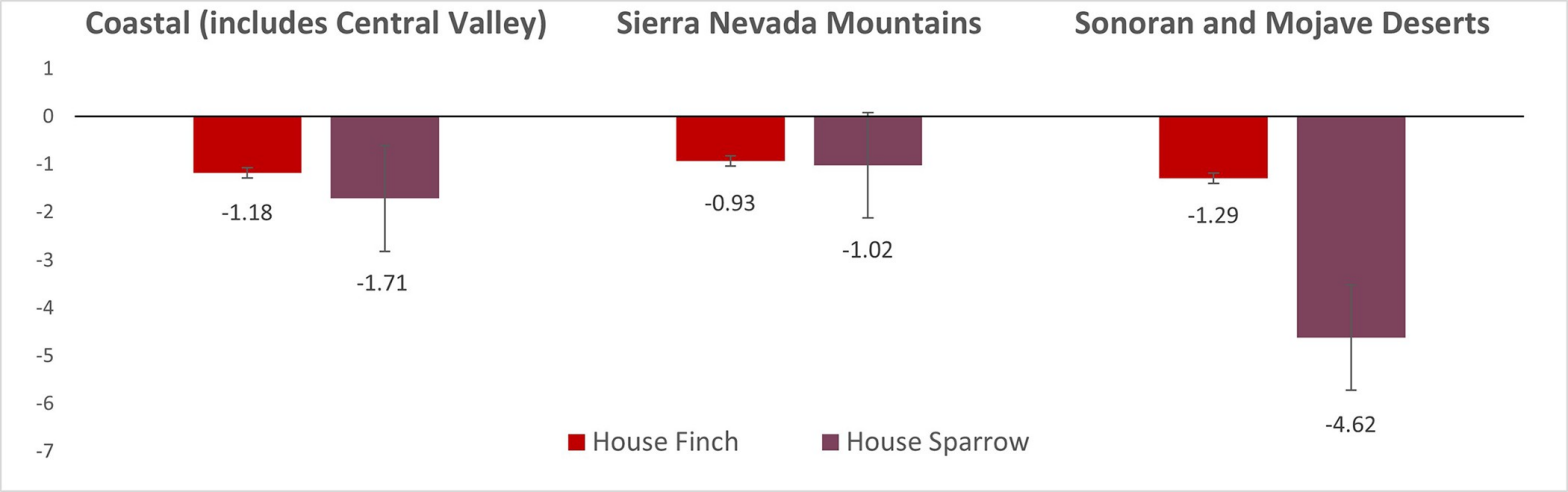
House Finch and House Sparrow population trends in three Bird Conservation Regions (2004–2019): Relative abundance change (%) per year and standard errors.

In both the large Coastal region, and the Sierra Nevada Mountain region, American Crows and California Scrub-jays decreased; Common Ravens increased in all three (Fig 1). Yellow-billed magpies inhabit only the Coastal region, where they decreased by -8.33% per year, and Steller’s Jays inhabit the Coastal and Sierra Nevada Mountain regions where they decreased by 1–2% per year. Confidence intervals indicated that American Crow and California Scrub-jay trends were only significant in the Coastal region; for the remaining corvid species, differences in all three regions were significant. House Finches and House Sparrows decreased in all three regions, but confidence intervals indicated that only House Sparrow decreases in the Coastal and Sonoran and Mojave Deserts regions were significant (Fig 2).

## Discussion

For nearly two decades, the reporting and testing of dead wild birds have contributed to California’s WNV surveillance program. Since the initial introduction of WNV into the state, the utility of dead birds in WNV surveillance has endured with program modifications despite fewer reports. In addition, bird mortality patterns from WNV infection appears to have changed from the early epidemic to recent endemic years in our study, which is evidence of a continually changing enzootic system.

### Program operations

Compared to 2005, when 82 (92%) of 92 VCAs participated in the DBSP, only 53 (60%) of 88 VCAs participated in 2020. In the interim 16 years, adjustments to protocols allowed for more cost-effective surveillance, by limiting collection criteria to the most susceptible bird species and submitting nucleic acid preservation cards from avian oral swab samples by regular mail rather than overnight shipping of carcasses for necropsy and tissue testing [37]. Increasing the age of carcasses acceptable for testing helped VCAs increase the sample size of testable birds; in addition, it is often difficult for the reporting individual to determine how long the bird has been dead. As an example, the Solano County Mosquito Abatement District broadened their collection criteria from dead <24h in 2019 to <48h in 2020. As a result, the number of birds submitted by the call center for pickup and testing in Solano County rose from 12 in 2019 to 28 in 2020. Of the 28 dead birds tested in 2020, 12 (43%) would have been rejected as unsuitable for testing by the call center because they were estimated to be dead longer than 24h, but less than 48h. There have been anecdotal incidents wherein a dried carcass (of unknown age) yielded a positive WNV result from an oral swab or maggot sample pressed onto a nucleic acid preservation card (LF pers. comm.). We also noted the COVID-19 pandemic did not appear to have a large impact on the dead bird surveillance program in 2020. Only two agencies halted or modified their dead bird programs due to COVID-19. All other agencies conducting dead bird surveillance in 2019 continued to operate dead bird surveillance programs in 2020, but with additional safety precautions [63].

Technology improved DBSP efficiency with smarter database algorithms to deliver tailored emails so that staff no longer needed to speak to each resident by telephone. This reduced missed calls/callbacks and saved time, especially during surge hours. In addition, the option for the public to attach a photo to their report allowed staff to better identify birds to type or species.

### Report volume by county

When WNV was first introduced into California, public awareness, concern, and media attention for WNV were at all-time highs [62]. In 2004, WNV was detected in at least one dead bird from each of the state’s 58 counties, the only year when WNV activity was documented in every county [62]. In the early years, high levels of media interest and public awareness were likely a major influence on this high number of dead bird reports and widespread WNV detections in dead birds. For example, in 2004 over 137,000 reports were received [62]. In 2018–2020, the public continued to report dead birds, but at lower frequencies consistent with the virus’s recurrent endemic status and less media attention; in 2020, 5,850 reports were received [47]. The top ten dead bird reporting counties in the later years were six Southern California or Central Valley counties, whereas the previously top reporting counties of San Bernardino, Riverside, Stanislaus, and San Joaquin counties were absent despite high levels of WNV activity in some of these areas (e.g., Stanislaus and San Joaquin counties). In San Bernardino and Riverside counties, decreased coverage for dead bird participation in the later years could have influenced report levels (participation in both counties went from two participating VCAs to one) [S1 Table].

Many of the top ten counties for dead bird reports in either time period had the greatest number of human WNV cases in 2009–2018 [64]: Fresno, Los Angeles, Orange, Riverside, Sacramento, San Bernardino, and Stanislaus. This suggests that dead bird reporting activity generally occurs in the same regions where human WNV cases occur, making dead birds a relevant WNV surveillance tool. Dead bird report numbers and report incidence did not generally correspond with areas of highest human case incidence, especially in endemic years 2018–2020, as areas with high human case incidence tend to be sparsely populated (e.g., Glenn County; 9). San Diego County’s low dead bird report numbers despite its large population size were likely due to the county’s relatively low WNV activity levels. Our results reinforce that urban residents find and report more dead birds, and as a result the DBSP has been more useful in detecting WNV activity to VCAs in urban versus rural areas. Researchers analyzing WNV surveillance data should be aware of this surveillance bias.

Generating dead bird reports from the public requires more effort today than during the initial surges of WNV in California due to waning public interest and engagement. Focus on invasive *Aedes* spp. mosquito reporting and bite/breeding prevention has diverted VCA and public attention. Although CDPH currently provides outreach messaging via social media [25] and press releases, the majority of public outreach comes from local VCAs. Dead bird reports often increase following a public messaging campaign or press release [65].

*Hypothesis 1*: Our first overarching hypothesis was that dead bird reports have decreased in later years due, in part, to decreases in bird populations. Dead corvid reports decreased disproportionately from early to later years compared to dead bird reports of most songbirds, raptors, and most other taxa, which was underscored by BBS decreases in corvid abundance from 2004 to 2019 (American Crows, California Scrub-jays, Yellow-billed Magpies, and Steller’s Jays). Common Ravens were the exception, with abundance increases in BBS data, corresponding to a less dramatic decline in dead Common Raven reports among the corvids in our study. These findings point to fewer corvids (except for Common Ravens) in California since the introduction of WNV.

However, regional population trends may not always reflect the overall trend. In at least one region, American Crow and Common Raven populations have been surging. San Francisco Bay Area counties have witnessed more and more of these birds roosting and foraging in urban areas for an unspecified number of years [66, 67]. This may be one reason several Bay Area counties appeared on the top 10 county list for dead bird reports in 2018–2020 (Alameda, Contra Costa, San Mateo, and Santa Clara). In 2019, the Audubon Society Christmas Bird Count (CBC) in Oakland (Alameda County) recorded all-time highs of American Crows [68], and in 2020, the CBC in Santa Clara County recorded all-time highs of American Crows and Common Ravens [69]. Abundance of corvids in this highly populated region likely contributed to higher dead bird report volume in four Bay Area counties in 2018–2020. Although some mortality can be attributed to WNV, American Crows also die of other causes including non-viral pathogens [29]. Looking ahead, although WNV activity in the Bay Area in 2018–2020 was mild compared to many of the years prior, robust corvid populations and warmer summers due to climate change may affect future WNV activity and the frequency of outbreaks in this region [70].

Breeding Bird Survey data also indicated a significant decrease in House Sparrows, House Finches, and American Robins from 2004 to 2019. However, House Sparrows decreased in abundance at more than twice the rate of House Finches. This complements our findings that dead sparrow reports (including other sparrow species) disproportionately decreased from the early to the later time periods. Although some mortality in sparrow species could be attributed to other causes (e.g. other diseases, changes in food abundance, and weather/climate conditions) [44] evidence that House Sparrows have declined since the introduction of WNV is strong, and since they are amplifying hosts, this may have implications for WNV ecology in Southern California where they drive WNV trends [16].

Although dead quail reports also decreased more than would be expected in later years (suggesting their numbers are now fewer), we did not examine these species further due to their tendency to survive WNV infection [46]. One study noted a decrease in California Quail *(Callipepla californica)* from 2004–5 to 2006–7 and suggested it may have been due to severe drought [71]. Thus, since quail are not highly susceptible to WNV, other factors are more likely to have impacted this species.

Other research also supports the idea that some avian populations have decreased since the early 2000’s due to WNV. Studies following WNV introduction in North America documented decreases in corvids, finches, sparrows, thrushes, and other bird species [44, 72–76]. Decreases in Yellow-billed Magpie reports alone (10,262 in the early period vs. 194 in the later period; Table 3) indicated this threatened corvid species has suffered further population declines since the introduction of WNV [73, 77]. However, another study found that many bird species in several U.S. states have recovered from the early impacts of the virus except American Crows, which now maintain a lower overall population mean compared to pre-WNV years [77]. Dead bird report volume in the DBSP has not previously been used as an indicator of avian population changes; however, it may serve as complementary data in population studies. Robust aggregate data such as Breeding Bird Survey [78] or Christmas Bird Count [79] usually provide the foundation for such studies.

### West Nile virus prevalence

A less dramatic drop in finch, sparrow, and thrush infection prevalence compared to corvids from the early to the later period is likely due to their role as reservoirs that do not always die from WNV infection [41, 42, 71]. Corvids are more susceptible to WNV and therefore more sensitive to fluctuations in WNV activity [38, 71]. Common Ravens, the only species to show no significant difference in WNV prevalence from the early to later years, may be less exposed than American Crows to WNV as they tend to inhabit forests, coastlines, and natural areas with less mosquito activity and/or humans to discover them [66, 79]. However, Common Ravens are increasingly moving into urban areas [66, 67], and proportionally increased over time in dead bird report data. Perhaps this species has experienced a release from competition with American Crows due to crow population declines, allowing ravens to expand their ranges into previously American Crow-dominated areas [77].

West Nile virus prevalence in tested dead raptors decreased considerably from the early to the later time period, perhaps due to overall lower WNV prevalence in prey and the environment [80]. The raptor species we studied also showed no indication of decreased populations in BBS data from 2004 to 2019; in fact, their populations may have slightly increased.

Another reason for lower WNV prevalence in tested dead birds as well as lower dead bird reports also might be due to less overall WNV activity, observed since 2017. Overall WNV prevalence in all tested dead birds in 2004–2006 was 56% (2004), 33% (2005), and 22% (2006) [9]; and in 2018–2020 it was 22% (2018), 12% (2019), and 20% (2020) [9, 47, 81]. Lower human WNV cases also have been reported in later years [9], such as 2018 when the lowest case count in California was reported (217 cases) [47]. Statewide mosquito minimum infection rates (minimum number of infected female mosquitoes per 1,000 tested) were also low (2.7–3.9) during 2018–2020, and these levels were not observed since the last dip in statewide WNV activity in 2009–2011 [8, 47]. While sentinel chicken seroconversions have also decreased, fewer flocks were deployed in recent years compared to 2004–2006 (253 flocks in 2004 vs. 95 flocks in 2020) which likely contributed to the decrease in total seroconversions [13, 47]. The recent lows in WNV activity have likely caused the report of fewer dead susceptible and moderately susceptible avian hosts. One other consideration is that perhaps after nearly two decades with WNV activity, VCAs are more experienced at pre-emptively targeting sources of mosquito production and controlling WNV-carrying mosquitoes, thereby effecting lower WNV activity in their jurisdictions.

*Hypothesis 2*: Our second hypothesis was that birds remain susceptible to dying from WNV, despite the length of time WNV has been endemic in California. The key avian species in our study did continue to test positive for WNV in the later years (although less so than in early years), which suggests they are still dying from WNV infection. Given the complexities of immunology, our study could not definitively prove nor disprove this hypothesis, but the question of whether or not birds are developing various levels of immunity to WNV continues to dominate avian WNV research. Evidence points to some capacity for immunity, as discussed below.

Bird immunity to viruses can develop by various modes and may not always be permanent. Studies showed House Sparrows can live with persistent lifelong infection, although it is not always detected in assays [82, 83]. In Southern California, House Sparrows and House Finches that survived WNV infection were seropositive for antibodies, thus creating heightened flock immunity dampening WNV activity in successive years [16]. However, as immune birds are rapidly replaced with immunologically naïve birds, herd immunity in these maintenance hosts wanes leading to a three-year amplification cycle with recurrent tangential transmission to humans [16]. In experimental studies, House Sparrows developed lower viremias over time in response to the NY99 strain; however, more recent WN02 and SW03 strains produced higher viremias in House Sparrows, pointing to an ongoing evolutionary “arms race” between this species and WNV [84]. American White Ibis chicks *(Eudocimus albus)* inherit maternal antibodies to WNV, but these antibodies wane rapidly over time [85]. West Nile virus immunity in other species such as corvids is not well understood or extensively studied. Although reports of American Crows recovering from WNV infection have been documented recently [86], they have continued to signal WNV activity in the DBSP, in agreement with the finding that current WNV strains WN02 and SW03 cause high mortality in corvids similar to the original introduced strain (NY99) in the U.S. [51]. A decline in most corvids and House Sparrow populations in recent years may indicate continued susceptibility; however, birds surviving infection probably develop permanent immunity, which helps to dampen WNV activity in the cyclic trends as already mentioned [87]. A separate question regarding the evolution of the more permanent adaptive, innate avian immunity, does not have sufficient evidence. In fact, the role of House Sparrows becomes clouded when considering their population declines from 2004 to 2019. This indicates House Sparrows may not have developed innate immunity or that new strains of WNV have retained/enhanced virulence for some avian populations. It would also suggest that House Finches may have developed some innate immunity because they did not decline at the same rate as House Sparrows during this same time span. More research is needed to better understand the mechanisms of avian immunity in response to WNV [49].

### First WNV indicator in county

Beginning with data from mid-April to the end of year, positive dead birds were the most common first indicator of WNV activity at the county scale in 2005, but positive mosquitoes were the most frequent first indicator in 2020. This may be due to a combination of decreased dead bird reports (due to fewer birds as discussed, as well as reduced public participation), modified VCA surveillance operations and new focus on invasive *Aedes* spp. mosquitoes in public messaging, and perhaps a biological reason in avian hosts. In addition, approximately 1.5 times more mosquitoes were tested in 2020 (1,304,457) [47] than in 2005 (870,485) [9], thereby increasing the chances of detecting low levels of mosquito infection. Detections in dead birds and sentinel chickens also occurred later in the WNV season in 2018–2020 compared to in 2004–2006. Whether or not this is a lasting trend remains to be determined; however, vector-borne disease research has pointed to the disruptive influence of anthropogenic changes (such as urbanization) and climate change on both vector and host phenology [88–91].

Bird species identification is one limitation of this study. Prior to 2006 when a SQL dead bird database was created, staff typed bird species by hand, resulting in ambiguous entries such as “jay.” With the new database, a dropdown menu solved this issue. Photos (mostly from smart phones) and bird identification applications have increasingly improved species determination; however, it is not always possible if the reporting individual cannot describe the bird or provide a photo, or if the carcass is in poor condition. Therefore, identification may be inaccurate in some reports and even in some tested birds. Carcasses also were identified by 1–2 technicians at CAHFS in the early years, whereas today they are identified by VCAs with varying identification skill levels and time dedicated to identifying birds. Another limitation is that public messaging may have focused on specific bird species in different areas and years, which could have influenced the species reported. Additionally, birds are just one component of a complex WNV zoonotic transmission cycle which also involves mosquito vectors, environmental conditions, and human activity. Our study did not include these other influential data (i.e., mosquito abundance and infectivity, climate, vector control measures).

Although considerable time and labor is needed to answer calls and online reports, coordinate pickups, and collect and test dead birds, dead bird surveillance is more cost-efficient than mosquito and sentinel chicken surveillance [24] based on data during 2004–2012. In addition, a recent analysis of the California Mosquito-Borne Virus Surveillance and Response Plan found that including all surveillance elements (dead birds, mosquitoes, and sentinel chickens) in risk assessment returned better predictive power for human incidence than vector index alone (a measure based on vector abundance and mosquito infection rate) [92], which corroborates other findings [18].

### Further research

Over the past two decades since WNV first arrived in California, avian data trends indicate evolving dynamics in this complex system. However, more research is needed to augment findings from earlier studies such as integration of local bird biology and behavior with *Culex spp*. vector host selection [93], wild bird serosurveys to measure antibody levels [17], estimate herd immunity and susceptibility [18, 94], and spatial analysis using all surveillance elements [55, 92]. Assessment of the changing WNV pathogenicity in key avian hosts [84] would also contribute to a better understanding of WNV transmission with the overarching goal to improve surveillance and control practices to further limit tangential transmission of WNV to humans.

## Conclusion

West Nile virus is the most important vector-borne disease in California, and it is critical to maintain a comprehensive WNV surveillance program to inform mosquito control practices and public health outreach. Strong collaboration among local VCAs, UC Davis, and CDPH is a primary reason dead bird surveillance has endured as part of California’s statewide WNV surveillance program, despite the decrease in reports. Although this study showed reduced utilization of dead birds as a surveillance tool in California since the early epidemic years, it also found evidence of population declines in important avian hosts and shifts in WNV seasonality. Dead bird surveillance remains important to better understand WNV ecology and evolution, and to add critical data to comprehensive environmental surveillance. Continued investment in testing dead birds, along with new strategies to increase public engagement would help ensure the dead bird surveillance program remains an effective surveillance tool.

## Supporting information

S1 Table Species and conditions of dead birds accepted by agencies in 2005, and in 2020. (PDF)

S2 Table Dead bird report totals by county for early years (2004–2006), later years (2018–2020), differences, and percent decreases. (PDF)
