# Supplementary material for: Epidemic versus endemic West Nile virus dead bird surveillance in California: Changes in sensitivity and focus
Source: PLoS One. 2023 Apr 6;18(4):e0284039. doi: 10.1371/journal.pone.0284039 (PMC10079120; doi:10.1371/journal.pone.0284039)
Supplement: S1 Table — (PDF) [file pone.0284039.s001.pdf]

**Agency**	**Accepted species/conditions**									
	**2005**	**2020**								
	**All species dead less than 24h, (excluding doves, pigeons, quail)**	**Dead less than 24h**	**Dead less than 48h**	**All species dead less than 24h, (excluding doves, pigeons, quail)**	**Corvids only (crows, ravens, jays, magpies)**	**Corvids, raptors only**	**Corvids, raptors, and songbirds only**	**Ants, trauma, maggots OK**	**NO shore/water birds, turkeys**	**Notes**
**Bay Area**										
Alameda Co. MAD	all	x	x	x				x	x	
Alameda Co. VCD	all	absorbed by Alameda MAD								
City of Berkeley EH (Alameda Co.)	unknown	absorbed by Alameda MAD								
Contra Costa Co. MVCD	all	x	x		x			x	x	
Marin-Sonoma Co. MVCD	all	x			x					Opened July 1
Monterey Co. EH	all	x			x					
Napa Co. MAD	all	x					x		x	Western half of county only
Northern Salinas MAD (Monterey Co.)	all	x				x				
San Benito Co. Agr. Comm.	all	x			x					
San Francisco Co. PH/Env. Health	all	x		x						
San Mateo Co. MVCD	all	x		x					x	
Santa Clara Co. VCD	all	x	x				x		x	
Santa Cruz Co. MVCD	all	x					x		x	
Solano Co. MAD	all	x	x				x			
**Northern Central Valley and mountains**										
Beale Air Force Base PH (Yuba Co.)	unknown	none								

Burney Basin MAD (Shasta Co.)	all	none								
Butte Co. MVCD	all	x		x						accepts pigeons, doves, quail; dead less than 24h
Colusa Co. MAD	all	x		x						
Durham MAD (Butte Co.)	crows and ravens only	absorbed by Butte MVCD								
El Dorado Co. EH	all	x		x						
El Dorado Co. VCP	all	x		x						
Glenn Co. MVCD	all	x		x						
Lake Co. VCD	all	x		x						
Nevada Co. EH	all	x		x					x	also excludes vultures
Oroville MAD (Butte Co.)	all	absorbed by Butte MVCD								
Pine Grove MAD (Shasta Co.)	all	none								
Placer Co. MVCD	all	x	x	x				x		accepts pigeons, doves, quail; dead longer than 48h
Sacramento-Yolo Co. MVCD	all	x	x	x				x		accepts doves, quail, but not pigeons; dead longer than 48h
Shasta Co. MVCD	all	x			x					
Sutter-Yuba Co. MVCD	all	x		x						
Tehama Co. MVCD	all	x		x						
**Southern San Joaquin**										
East Side MAD (Stanislaus Co.)	all	x	x		x					

Merced Co. MAD	all	x		x						Opened May 1
San Joaquin Co. MVCD	all	x	x	x						
Turlock MAD (Stanislaus Co.)	all	x		x						
**Central Valley**										
Coalinga-Huron MAD (Fresno Co.)	unknown	none								
Consolidated MAD (Fresno Co.)	all	none								
Delano MAD (Kern Co.)	all	none								
Delta VCD (Tulare Co.)	all	x	x				x		x	also accepts pet birds and hummingbirds; dead greater than 48h
Edwards Air Force Base (Kern Co.)	all	none								
Fresno Co. MVCD	all	x		x						
Fresno Westside MAD (Fresno Co.)	all	x		x						
Kern Co. EH	all	none								
Kern Co. MVCD	all	x				x				
Kings Co. MAD	all	none								
Madera Co. MVCD	all	x			x					
South Fork MAD (Kern Co.)	unknown	none								
Tulare Co. MAD	all	x		x						
West Side MVCD (Kern Co.)	all	none								
**Los Angeles County**										
Antelope Valley MVCD	all	x	x			x		x		
City of Pasadena PH Dept.	all	none								
Compton Creek MAD	unknown	x	x		x (crows and ravens only)					
Greater LA County VCD	all	x		x						

Long Beach Health Dept. and VCP	all	x			x					Opened May 13
Los Angeles West VCD	all	x			x					
Los Angeles Co. Veterinarian	all	none								
San Gabriel Valley MVCD	all	x		x						
**Southern California**										
Camp Pendleton Preventative Medicine Dept. (San Diego Co.)	unknown	none								
City of Moorpark VC (Ventura Co.)	all	x		x					x	
Coachella Valley MVCD (Riverside Co.)	all	x		x						
Imperial Co. EH	all	none								
Imperial Co. VC	all	none								
MVCD of Santa Barbara Co.	all	x					x		x	
Northwest MVCD (Riverside Co.)	all	x	x	x				x		
Orange Co. MVCD	all	closed (but tested a number of birds)								
Riverside Co. EH	all	none								
San Bernardino Co. MVCP	all	x					x		x	
San Diego Co. VCP	all	x				x			x	
San Luis Obispo Co. PH Lab	all	x	x	x						
Vandenberg Air Force Base (Santa Barbara Co.)	unknown	none								
Ventura Co. EH	all	x					x		x	
West Valley MVCD (San Bernardino Co.)	all	x		x					x	no pet birds
**Remaining**										
Alpine Co. Health Dept.	unknown	none (although 2 birds were tested)								

Amador Co. Agr. Comm.	all	none								
Calaveras Co. EH	all	none								
Del Norte Co. Health Dept.	all	none								
Humboldt Co. EH	all	x					x		x	
Inyo Co. EH	all	none								
Lassen Co. EH	all	none								
Mammoth Lakes MAD (Mono Co.)	all	none								
Mariposa Co. Health Dept.	all	x		x						
Mendocino Co. EH	all	none								
Modoc Co. Health Dept.	all	none								
Mono Co. EH	all	none								
Owens Valley MAP (Inyo Co.)	all	none								
Plumas Co. EH	all	none								
Saddle Creek Community Services District (Calaveras Co.)	all	none								
Sierra Co. EH	all	none								
Siskyou Co. EH	all	none								
Trinity Co. EH	all	x	x	x						
Tuolumne Co. EH	all	x		x						
Yosemite National Park Division of Resources Mgmt & Science (Mariposa and Tuolumne Co.)	unknown	none								
