# Supplementary material for: Epidemic versus endemic West Nile virus dead bird surveillance in California: Changes in sensitivity and focus
Source: PLoS One. 2023 Apr 6;18(4):e0284039. doi: 10.1371/journal.pone.0284039 (PMC10079120; doi:10.1371/journal.pone.0284039)
Supplement: S2 Table — (PDF) [file pone.0284039.s002.pdf]

County	2004-2006 totals	2018-2020 totals	difference	% decrease
San Mateo	2,808	886	1,922	68
Orange	6,514	1,673	4,841	74
Santa Clara	7,925	1,628	6,297	79
Santa Cruz	1,636	305	1,331	81
Alameda	5,396	936	4,460	83
San Diego	3,283	558	2,725	83
Unknown	780	131	649	83
San Francisco	1,065	154	911	86
Yolo	4,675	645	4,030	86
Contra Costa	11,528	1,533	9,995	87
Yuba	803	103	700	87
Sacramento	25,121	2,989	22,132	88
Placer	7,212	787	6,425	89
Sutter	1,593	162	1,431	90
Humboldt	529	39	490	93
Los Angeles	33,599	2,395	31,204	93
Napa	833	57	776	93
Colusa	225	15	210	93
Tuolomne	439	29	410	93
Alpine	31	2	29	94
El Dorado	2,722	172	2,550	94
Solano	4,396	272	4,124	94
San Joaquin	8,293	504	7,789	94
Ventura	4,118	250	3,868	94
Tulare	4,365	250	4,115	94
Butte	3,898	217	3,681	94
Fresno	12,911	700	12,211	95
Marin	2,783	144	2,639	95
Stanislaus	9,421	463	8,958	95
Monterey	1,554	74	1,480	95
Merced	3,688	168	3,520	95
Sonoma	5,250	229	5,021	96
Lake	1,033	45	988	96
Del Norte	70	3	67	96
Riverside	10,397	432	9,965	96
San Benito	337	14	323	96
Santa Barbara	1,948	80	1,868	96
Madera	970	38	932	96
Amador	640	24	616	96
Nevada	1,441	54	1,387	96

Shasta	2,129	78	2,051	96
Inyo	494	16	478	97
Imperial	97	3	94	97
Sierra	33	1	32	97
San Luis Obispo	2,313	68	2,245	97
Tehama	1,013	29	984	97
Trinity	74	2	72	97
San Bernardino	12,531	326	12,205	97
Kern	3,136	79	3,057	97
Mendocino	960	19	941	98
Kings	1,970	38	1,932	98
Plumas	313	6	307	98
Calaveras	1,006	17	989	98
Glenn	627	10	617	98
Mono	188	2	186	99
Lassen	189	2	187	99
Siskiyou	206	2	204	99
Mariposa	274	1	273	100
Modoc	72	0	72	100
Grand Total	223,855	19,859	203,996	91
